# Estimating Maternal Mortality in Remote Rural Regions: an Application of the Sisterhood Method in Tajikistan

**DOI:** 10.5195/cajgh.2019.341

**Published:** 2019-01-23

**Authors:** Kylea Laina Liese, Heather Pauls, Sarah Robinson, Crystal Patil

**Affiliations:** 1 Department of Women, Children, and Family Health Sciences, University of Illinois, Chicago, USA; 2 Office of Research Facilitation, University of Illinois, Chicago, USA; 3 Department of Psychology and Educational Sciences, University of Geneva, Switzerland

**Keywords:** Maternal Mortality, Sisterhood Method, Tajikistan, Gorno-Badakhshan Autonomous Oblast

## Abstract

**Introduction:**

The sisterhood method of maternal mortality data collection and analysis provides a validated framework for estimating maternal mortality ratios in situations of limited infrastructure. The aim of this study is to assess sub-national maternal mortality in the Badakhshan region of Tajikistan using the sisterhood method as part of a larger ethnographic study on maternal risk.

**Methods:**

In 2006–2007, 1004 married women of reproductive age in Gorno-Badakhshan Autonomous Oblast, Tajikistan were surveyed using the sisterhood method. Respondents were asked eleven questions about the sex, age and survivorship of all children born to the respondent’s mother.

**Results:**

Using a national total fertility rate (TFR) estimate of 4.88, the maternal mortality ratio (MMR) in Tajik Badakhshan was 141 maternal deaths per 100,000 live births (95% CI 49–235). The lifetime risk of maternal death was 1 in 141 (95% CI 34–103).

**Conclusion:**

Given the inherent time-lag of the sisterhood method, precise estimates of maternal mortality are dependent on accurate TFRs, which may vary based upon regional experiences of demographic transitions. Socio-political instability and the dismantling of Soviet welfare programs and civil war following Tajikistan’s independence from the Soviet Union in 1991 likely impacted TFR in Tajik Badakhshan. Socio-political trends influencing TFR in rural regions compared to urban, and the investigation of factors associated with maternal mortality, require additional investigation.

## Introduction

Countries with the highest rates of maternal mortality typically do not have strong health information systems. These systems are vital to generate accurate maternal mortality ratio (MMR), the statistical backbone on which millions of dollars are spent through research, programming, monitoring, and evaluation. However, maternal mortality ratios are complex indicators with estimation requiring both the accurate identification of cause of death and large sample sizes.^1^–^2^ The sisterhood method of maternal mortality data collection and analysis provides a validated framework for estimating maternal mortality ratios in situations of limited infrastructure.^2^–^3^ The sisterhood method embedded in Demographic and Health Surveys (DHS) produce maternal mortality ratios for at least 28 countries, comprising 16% of global births.^3^ With better estimates of maternal mortality at the subnational level, health care spending could be tailored to address the specific needs of rural populations.

Maternal mortality rates in post-Soviet Central Asia are lower than those in countries with similar income levels, likely due to comprehensive social and economic development programs of the Soviet era (e.g., paved roads, compulsory education, access to healthcare and minimum marriage age). In Tajikistan, the poorest of the post-Soviet countries, health care in the mountainous region of Gorno-Badakhshan Autonomous Oblast (administrative region) (GBAO) is limited compared to urban centers. Although GBAO makes up 45% of the land of Tajikistan, it is scarcely populated with only 218,000 residents (3% of the national population).^5^ Villages in GBAO are situated along tributaries leading into and along the Pyanj River, the border between Tajikistan and Afghanistan. As citizens of a former Soviet republic (1929–1991), Tajik women in GBAO had greater access to quality obstetric care and were rewarded for having upwards of ten offspring to grow “The Fatherland.” However, with the breakdown of Soviet Union in 1991, women were encouraged to curb their fertility.^6^–^7^ The dismantling of Soviet welfare programs and the socio-political instability associated with independence contributed to a civil war between 1992–1997, which further destabilized the country’s health and social infrastructure. This transition directly and indirectly impacted maternal risk as women’s access to high quality obstetric care declined, and funding for maintaining roads, schools, and hospitals was reduced.

The data presented here come from a maternal mortality survey using the sisterhood method^2^ conducted in the Darwaz District of GBAO Tajikistan in 2006. This survey was part of a larger anthropological study that used mixed-methodologies to explore the underlying bio-social context of maternal risk in the Badakhshan regions shared between Tajikistan and Afghanistan. In 2001, Afghan Badakhshan was reported to have the highest maternal mortality ratio ever recorded (6507 deaths per 100,000 live births) in a maternal mortality survey that utilized the sisterhood method.^8^ Although national estimates suggested that maternal mortality was much lower in Tajik Badakhshan, situated directly across the Pyanj River, no subnational data existed. Since these regions share important ethnic and geopolitical features (e.g., poor roads, mountainous terrain, and histories of civil war and drug trade), investigating GBAO maternal mortality rates is very important.

The purpose of this study was to obtain an estimate of maternal mortality in the Badakhshan region of Tajikistan and to compare it with Afghan Badakhshan, harnessing previously collected comparative ethnographic data.

## Materials and methods

This study was carried out in 2006–2007 in GBAO. With a population of 23,600, Darwaz District was selected because of a bridge connecting villages on either side of the border, permitting the lead researcher a unique regular access to otherwise isolated Afghan and Tajik villages. The population of Darwaz is predominantly ethnically Tajik and religiously Muslim. In addition to the survey, the study included semi-structured interviews (n=184) with married women of reproductive age, which collected observations on birth and reproductive health care in local clinics and hospitals. Also included were key informant interviews with community and religious leaders and healthcare providers in Tajik and Afghan villages situated opposite each other along the border.

After training, three local female field assistants fluent in Tajiki language conducted household surveys, recording the data using the standardized validated forms. Over the course of 7 months, every third household in 24 villages in Tajik Badakhshan was selected and visited. The villages were selected based on population (< 300 households, > 300 households), geographic location (road access, along a river/tributary, on a mountainside), and distance from the district hospital in Kalai-Khumb. All women present in the household who were 18 and older and who were not sisters were invited to participate. A total of 820 participants answered the minimum four interview questions necessary for the analysis. This short survey was also embedded in a longer semi-structured interview for another 184 participants, resulting in a total of 1004 participants. The longer ethnographic interview instrument elicited a complete reproductive history, including onset of sexual activity, contraceptive use, details on each pregnancy and birth, and personal perspectives on the issues of gender, marriage, and childbearing risk in their village. No males were interviewed. Three subjects with unknown ages were excluded from the long interview group, resulting in the final sample size of 1001 research participants. This study was approved by the Stanford University IRB, and all respondents verbally consented to participating in the study.

By interviewing women about the survival of their adult sisters, the sisterhood method allows for retrospective maternal mortality estimates in remote regions like GBAO because it reduces sample size requirements and costs. The WHO has relied on maternal mortality estimates collected via the sisterhood method to provide crucial maternal health programming in the absence of vital registration systems since the 1980’s.^4^ According to the procedure for the direct sisterhood method (S1),^2^ respondents were asked eleven questions about the sex, age and survivorship of all children born to the respondent’s mother. For all sisters reported to be deceased who were married, respondents were asked for the year of death, age at death, and whether the sister died while pregnant, in childbirth, or within the 42 days following pregnancy or childbirth. Answers to these questions were used to obtain data on four indicators: 1) the number of sisters born to the same mother who reached the age of 15 years or older; 2) how many of these sisters were still alive; 3) how many of these sisters died; and 4) of those who died, how many died during pregnancy or within 42 days of birth. Undergraduate research assistants from the University of Central Asia in Dushanbe entered the data into an Excel database. Data were cleaned prior to analysis by reviewing and comparing the data in the excel spreadshees and the paper forms. Erroneous values and missing data were coded as missing (less than 2%).

Although the legal age of marriage in Tajikistan is 17, women regularly married at younger ages.^7^ To consider these cultural factors, we followed the method of Smith et al^9^ and counted every female sibling over the age of 15 as married. Siblings under the age of 15 were excluded, but their data were used to compute total reported siblings. Polygyny is illegal in Tajikistan and not routinely practiced. No homes with multiple wives were visited.

**Figure 1:**
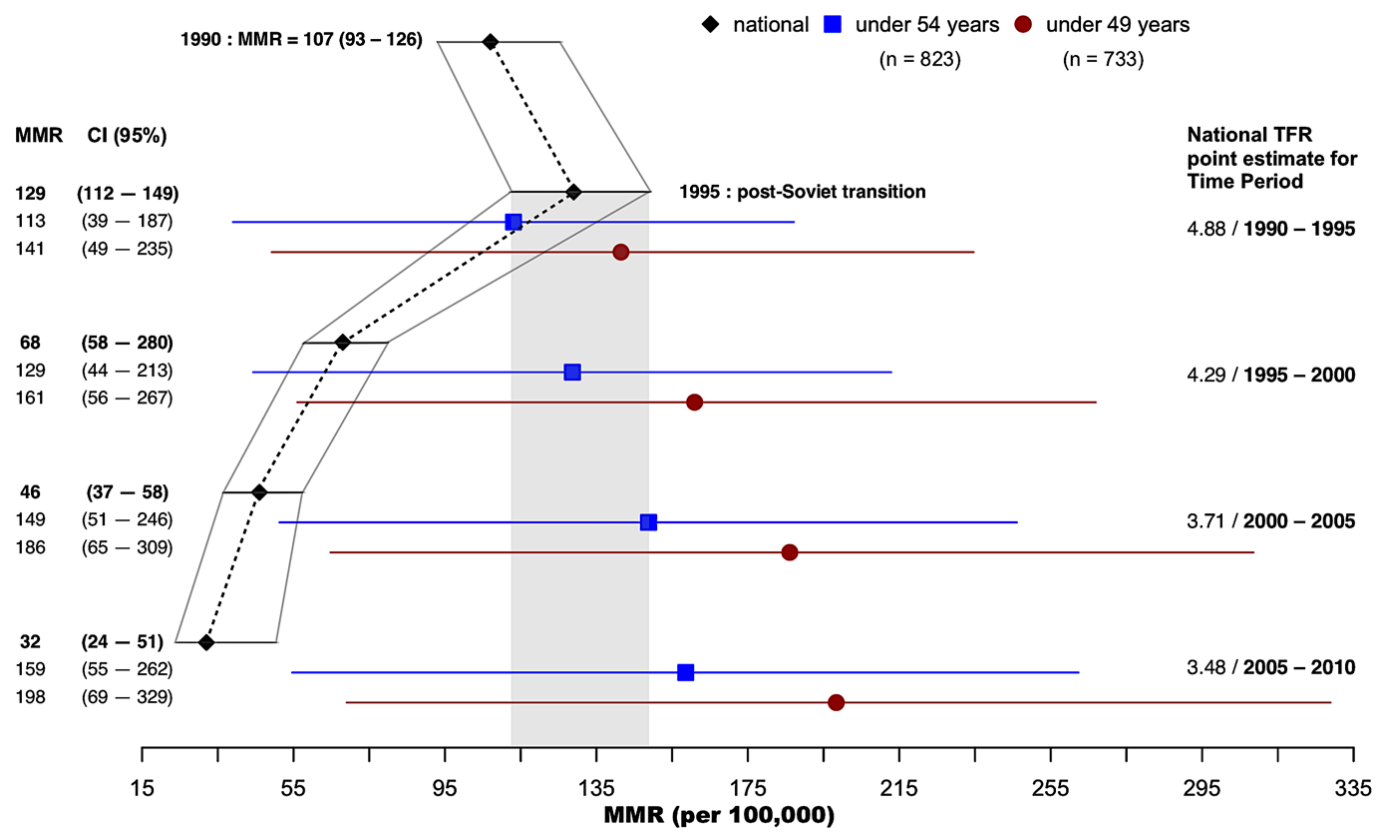
GBAO Tajikistan Sisterhood estimated MMR (per 100,000) with 95% confidence intervals MMR estimates using the national TFR (4.88) for the same period based on time-lag (1990–1995) are congruent with the spike shown for national levels in 1995. MMR estimates using dropping TFR values during the post-Soviet fertility transition illustrate the stability or potential increase in MMR in this region compared to the national estimates for the same time period if urban improvements in maternal mortality outpace rural.

For the 820 participants who completed the short survey, the respondent’s exact age was not collected. Respondent age was estimated, and age categories were assigned using birth order and reported sibling ages. To estimate respondent age, the population mean interbirth interval, the total age range of siblings divided by total number of siblings (IBI = 2.78), was added to the age of the sibling born just prior to the respondent. For the 181 women who received more in-depth interviews as part of the larger ethnographic study, year of birth was collected, and we therefore used exact age. Five groups were used to produce age category estimates. We used R (2018) version “Joy in Playing” for calculations based on the methodology suggested by Graham [Table 1].^2^ Confidence intervals (95%) were calculated for lifetime risk [Table 2] and MMR [Figure 1] using the method published by Hanley.^6^

The lifetime risk of maternal death was calculated by dividing the number of maternal deaths by the sister units of risk exposure, q(w) = r_i_B_i_/, where the sister units of risk exposure was given by the number of ever-married sisters multiplied by an adjustment factor, B_i_ = N_i_A_i_. Adjustment factors are corrections based on a found age-distributed pattern between the proportion of sisters dying of maternity related causes and the probability of dying from those causes.^2^ We also applied a correction to the number of sisters at risk in the younger respondent group by multiplying the number of respondents by the average number of sisters reported by older respondents (25 and older). This adjusts for under-estimation of the number of total lifetime sisters for young respondents.^3^ Finally, because sample size was too small to rely on age group estimates, the data were aggregated over all age groups to give the total lifetime risk of maternal death, Q =∑r_i_∑B_i_/[Table 1 and Table 2]. To reduce the impact of potential reporting biases, we also followed Graham et al^2^ in restricting respondent age to 49 and under, however, a maternal death of a sister is a very memorable event,^7^ and we had no reason to believe that the memories of these older respondents were impaired.

The Maternal Mortality Ratio (MMR), is a function of the lifetime mortality risk and the total fertility rate, MMR = 1 - P^1/TFR^, where P = 1 - Q, the lifetime probability of avoiding death from maternal causes, and TFR is the total fertility rate, the total number of children born per woman in her lifetime, or likely to be, if exposed to current rates of age-specific fertility. When lifetime cumulative risk, Q, remains constant or increases in tandem with lower TFRs, the MMR increases, showing an increased risk of dying per pregnancy.

Standard errors for Q were calculated as ${\text{SE}}_{\text{Q}}=\sqrt{(\text{r}/\text{B})*1-(\text{r}/\text{B})/\text{B}}$. Upper and lower confidence limits of 95%, Q ±1.96(SE), were then substituted into the MMR equation to give upper and lower bounds on these estimates.^6^ Time-lags using this method^2^ also place aggregated estimates of MMR for ≤49 about 12 years before data collection. For our sample, the MMR then refers to the post-soviet transitional period, approximately 1994 – 1995.

## Results

The average number of siblings (brothers and sisters) reported was M = 6.5 (SD = 3.0) with a maximum of 23, however 93% of respondents reported 10 or less siblings. An average of M = 4.95 (SD = 2.26) sisters with a maximum of 11 was found. Results for each age group are reported in Table 1. For all respondents, the ever-married number of sisters was N = 3429, with 103 reported as deceased (15/103 mortality events attributed to maternal causes).

We report both the total lifetime risk, Q, for population age groups ≤49 and for ≤54 [Table 2]. For Tajik Badkhstan using all respondents, Q = 0.0068, 95% CI [0.0034, 0.0103], or a lifetime risk of maternal death of 1 in 147. Lifetime risk of maternal death for age 49 and under is Q = 0.0069, 95% CI [0.0024, 0.0114], or 1 in 145. The lifetime risk for those 54 and under is 0.0055, 95% CI [0.0019, 0.0091], or 1 in 182.

The national TFR estimate for 1994–1995 is 4.88.^8^ Using this TFR, our estimate of the maternal mortality ratio (MMR) in Tajik Badakhshan is 141 maternal deaths per 100,000 live births, 95% CI [49, 235]. This figure is congruent with the MMR national estimate for that time frame, MMR = 129, CI [112, 149]^9^ represented in Figure 1 by the grey shaded region, matching the spike in MMR experienced nationally in Tajikistan in the immediate post-Soviet transition. This confirms the usability of MMR best estimates using the sisterhood method, despite small sample sizes and large confidence intervals. Our research also suggests the need for local estimates of TFR and the need for repeated studies to track MMR trends in rural areas.

As can be seen in Figure 1, MMR is highly dependent on TFR. Urban areas typically have lower fertility compared to rural regions, and the fertility transition is typically slower in these areas. While fast rates of fertility transition are similar across urban contexts, the diffusion to non-urban areas is, in contrast, characterized by large heterogeneity.^10^ In this study, 62% of respondents reported using some form of birth control, but given the limited ability to estimate TFR directly using the current data, we relied on the national TFR point estimate. For comparison, we also computed the MMR using the national TFR for the three following five-year periods (1995–2000, 2000–2005, 2005–2010), and contrasted these results with those given by WHO, UNICEF, UNFPA, World Bank Group, and the United Nations Population Division.^9^ [Figure 1]

## Discussion

We used the sisterhood method to produce a sub-national estimate of maternal mortality, MMR, for an isolated region in Tajikistan. The estimates we provide (using a range of national TFR estimates for four-time periods) refer to a period about 12 years before the survey, approximately 1994 – 1995. WHO, UNICEF, UNFPA, World Bank Group, and the United Nations Population Division (2015) found an MMR 129, CI [112, 149] for this timeframe, demonstrating a spike in maternal mortality during the post-Soviet transition.^12^ This estimate is higher than maternal mortality ratios in neighboring post-Soviet countries such as Uzbekistan (MMR 54) and Kazakhshan (MMR 91), though similar to Kyrgyzstan (MMR 120).^12^

Our result for GBAO is congruent with the national Tajikistan estimate, MMR = 141, CI [49, 235]. We suspected that the MMR for GBAO would be higher than the national MMR given its isolation as a semi-autonomous region in the Pamir mountains along the border with Afghanistan. Despite the stabilizing power of socialized health care during the Soviet era, emergency obstetric care was not always available in the region, though skilled midwives did attend most births.^7^ The post-Soviet transition involved dismantling key socialist welfare programs that contributed to conditions that increased maternal mortality risk nationwide.

There are multiple limitations to consider in this study. Though recall bias may be unlikely because a sister’s death is a significant and memorable socio-cultural event, it is possible some respondents failed to report maternal deaths of sisters who may have moved out of the area, resulting in an underestimation of maternal deaths. Recall bias may have also produced an overestimation of maternal deaths if some respondents incorrectly attributed deaths of sisters to maternal factors. It is possible that sisters living in the same village produced duplicated accounts. However, in this patrilineal society, women move to their husband’s village when they are married, making this limitation unlikely or uncommon.

The results are also potentially impacted by the lack of a local estimate of TFR during a period of dropping fertility nationwide. Figure 1 shows the effect of TFR estimates on estimates of the MMR. TFR decreases after the Soviet period ended (approximately 1991), yet the MMR spikes nationwide before quickly dropping. This is likely a reflection of the dramatic socio-political instability which followed independence from the Soviet Union. Tajikistan was not only cut off from Soviet benefits such as nutritional supplies, roads, and hospital supplies, but also plummeted into a lengthy civil war between 1992 and 1997, making it unique among the former Soviet countries of Central Asia. Cut off from the capital and bordering Afghanistan, GBAO was particularly impacted by the upheaval of the civil war and its aftermath.

However, a lower TFR is likely applicable to respondents in our study, rather than the TFR of their parents during the decades when the Soviets enforced pro-natal agendas in GBAO, which increased fertility substantially. The Tajikistan Demographic and Health Survey suggests that the region-specific TFR is 3.30, close to the 2005–2010 United Nations (2015) estimate (3.48). A lower TFR increases the MMR if individuals continue to be exposed to the 1994–1995 risk profile. This problem could be exacerbated as inequality between urban and rural areas often widens in periods of economic transition; maternal mortality may improve in urban areas but worsen in rural, requiring further investigation. These aspects of fertility and mortality require investigation. For this population, using national TFRs based on model life tables (as we do) may be a more reliable approach due to small sample sizes, but fertility transitions make applicability to rural areas uncertain. Therefore, estimating TFR locally while incorporating the uncertainty in this calculation could improve our grasp of region specific needs in these contexts. Discussion of important trends in TFR in rural regions compared to urban and the investigation of associated impacts on maternal mortality requires repeated studies of both local TFR and maternal mortality risk. This point is especially important to consider in similar post-Soviet countries of Central Asia where the collapse of the Soviet Union heightened health and social disparities between rural and urban populations.

Regional variation in maternal mortality may be as high as variation across international borders, necessitating targeted interventions to improve maternal outcomes at the local level.^9^ Global health efforts to reach developed countries’ standards may have greater impact when subnational variation in maternal mortality is addressed. Our results indicate that where social, political, and economic conditions contribute to rapidly changing mortality and fertility rates in small subnational populations, it is crucial that researchers use effective and accessible methods to estimate local TFR in order to better understand variation in maternal health outcomes at the local level. For example, the fall of the Soviet Union changed women’s lives in GBAO in ways that directly impact maternal risk, including education, marriage age, medical education, availability of medical supplies and contraceptives, hospital infrastructure, and accessibility.^7^ Understanding trends in maternal mortality in subnational areas in relation to those found using national metrics will require repeated studies.

## Figures and Tables

**Table 1:** Estimation of maternal mortality using the Sisterhood method for GBAO Tajikistan, 2006.

Age group of respondents, *i* (a)	Number of Respondents (b)	Sisters Ever-married, *N_i_* (c)	Maternal deaths, *r_i_* (d)	Adjustment factor, *A_i_* (e)	Sister-units of risk exposure, *B_i_* (f = ce)	Life-time risk of maternal death, *q*(*w*) (g = d/f)	Proportion of dead sisters dying of maternal causes (h)
18–24	173	593 *	2	0.151	90	0.0222	0.4000
25–34	254	807	4	0.421	340	0.0118	0.2222
35–44	208	798	2	0.737	588	0.0034	0.1429
45–54	188	659	1	0.934	616	0.0016	0.0500
55+	178	572	6	0.992	567	0.0106	0.1304
**Total**	**1001**	**429**	**15**	**--**	**2201**	**0.0068** ** **(1 in 147)**	**--**

* Derived for age 18–24 by multiplying the # of respondents 18–24 (n=173) by the average number of ever married sisters per respondent for age groups 25+ (3.425). This corrects for under-estimation of the number of total sisters for young respondents (Graham, 1989). Reported number of ever married for respondents 18–24 is 358.

** Lifetime risk of maternal death for respondents under 54 years of age = 9/1634 = 0.0055 (1 in 182) n = 823.

**Table 2:** Lifetime risk of maternal death

	*Q*, Lifetime Risk [CI]	
**All women**	0.0068 [0.0034 – 0.0103]	1 in 147 [1/294 to 1/97]
**≤49 years**	0.0069 [0.0024 – 0.0114]	1 in 145 [1/417 to 1/88]
**≤54 years**	0.0055 [0.0019 – 0.0091]	1 in 182 [1/526 to 1/110]

## References

[1] Blencowe H, Calvert C, Lawn JE, Cousens S, Campbell OM (2016). Measuring maternal, foetal and neonatal mortality: Challenges and solutions. Best practice & research Clinical obstetrics & gynaecology.

[2] Graham W, Brass W, Snow RW (1989). Estimating maternal mortality: the sisterhood method. Studies in family planning.

[3] Hill Kenneth (2007). Estimates of maternal mortality worldwide between 1990 and 2005: an assessment of available data. The Lancet.

[4] Shiffman Jeremy (2000). Can poor countries surmount high maternal mortality?. Studies in family planning.

[5] (2008). Population of the Republic of Tajikistan as of 1 January 2008.

[6] Khalid A (2007). Islam after Communism: Religion and Politics in Central Asia.

[7] Liese KL (2009). Motherdeath in Childbirth: Explaining maternal mortality on the roof of the world.

[8] Bartlett LA (2005). Where giving birth is a forecast of death: maternal mortality in four districts of Afghanistan, 1999–2002. The Lancet.

[9] Smith JB, Fortney JA, Wong E, Amatya R, Coleman NA, Johnson JDG (2001). Estimates of the maternal mortality ratio in two districts of the Brong-Ahafo region, Ghana. Bulletin of the World Health Organization.

[10] Hanley JA, Hagen CA, Shiferaw T (1996). Confidence intervals and sample-size calculations for the sisterhood method of estimating maternal mortality. Studies in Family Planning.

[11] Graham WJ, Foster LB, Davidson L, Hauke E, Campbell OM (2008). Measuring progress in reducing maternal mortality. Best practice & research Clinical obstetrics & gynaecology.

[12] United Nations, Department of Economic and Social Affairs, Population Division (2015). World Population Prospects: The 2015 Revision.

[13] World Health Organization (2015). Trends in Maternal Mortality 1990 to 2015 Estimates by WHO, UNICEF, UNFPA, World Bank Group and the United Nations Population Division.

